# Neuroimaging Techniques in Differentiating Parkinson’s Disease from Drug-Induced Parkinsonism: A Comprehensive Review

**DOI:** 10.3390/clinpract13060128

**Published:** 2023-11-15

**Authors:** Jamir Pitton Rissardo, Ana Letícia Fornari Caprara

**Affiliations:** 1 Neurology Department, Cooper University Hospital, Camden, NJ 08103, USA; 2 Medicine Department, Federal University of Santa Maria (UFSM), Santa Maria 97105-900, RS, Brazil; ana.fornari@acad.ufsm.br

**Keywords:** dopamine transporter, DAT, dopaminergic imaging, DIP, PET, SPECT, SWEED, SWIDD, neurotransmitter, drug-induced movement disorder

## Abstract

Neuroimaging can provide significant benefits in evaluating patients with movement disorders associated with drugs. This literature review describes neuroimaging techniques performed to distinguish Parkinson’s disease from drug-induced parkinsonism. The dopaminergic radiotracers already reported to assess patients with drug-induced parkinsonism are [123I]-FP-CIT, [123I]-β-CIT, [99mTc]-TRODAT-1, [18F]-DOPA, [18F]-AV-133, and [18F]-FP-CIT. The most studied one and the one with the highest number of publications is [123I]-FP-CIT. Fludeoxyglucose (18F) revealed a specific pattern that could predict individuals susceptible to developing drug-induced parkinsonism. Another scintigraphy method is [123I]-MIBG cardiac imaging, in which a relationship between abnormal cardiac imaging and normal dopamine transporter imaging was associated with a progression to degenerative disease in individuals with drug-induced parkinsonism. Structural brain magnetic resonance imaging can be used to assess the striatal region. A transcranial ultrasound is a non-invasive method with significant benefits regarding costs and availability. Optic coherence tomography only showed abnormalities in the late phase of Parkinson’s disease, so no benefit in distinguishing early-phase Parkinson’s disease and drug-induced parkinsonism was found. Most methods demonstrated a high specificity in differentiating degenerative from non-degenerative conditions, but the sensitivity widely varied in the studies. An algorithm was designed based on clinical manifestations, neuroimaging, and drug dose adjustment to assist in the management of patients with drug-induced parkinsonism.

## 1. Introduction

Drug-induced movement disorders impact a significant portion of the population. At least one percent of the population is estimated to suffer from tremors or ataxia secondary to medications [1,2]. In this context, there is a significant burden related to the high cost of extensive diagnostic workup, hospitalization, increased healthcare expenditures, and lost workdays due to drug-induced movement disorders. More than five percent of subjects initially presenting with Parkinson’s disease are commonly later diagnosed with drug-induced parkinsonism [3].

Neuroleptics are the most common class of medications associated with drug-induced parkinsonism [4]. They may block dopaminergic D2 receptors in the postsynaptic neurons. The prevalence of drug-induced parkinsonism in individuals managed with neuroleptics in the literature widely varies from 15% to 60% [2,5]. In this context, the duration of neuroleptic therapy, neuroleptic doses, and genetic predisposition of individuals may significantly influence the development of drug-induced parkinsonism [6]. Drug-induced parkinsonism can occur secondary to many agents, including antibiotics, antidepressants, antiseizure medications, and calcium channel blockers [7,8].

An inaccurate or delayed diagnosis of drug-induced parkinsonism may result in ineffective treatment and expose patients to side effects, impacting their quality of life. In this context, the clinical differentiation between Parkinson’s disease and drug-induced parkinsonism usually requires discontinuing the offending medication for a long period, which is often challenging and, in some cases, not feasible, such as in active neuropsychiatric disorders. Moreover, a levodopa trial could be effective in suspected subclinical parkinsonism, especially in parkinsonism secondary to dopamine-blocking agents [9].

**Figure 1:**
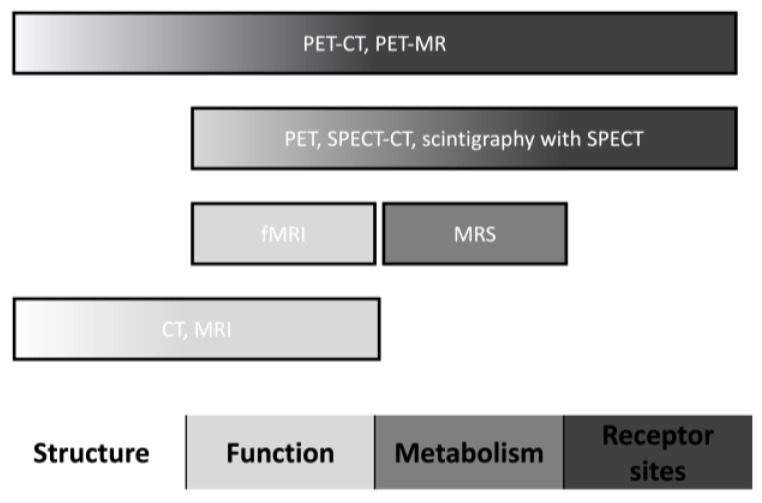
Neuroimaging characteristics. Abbreviations: CT, computed tomography; fMRI, functional magnetic resonance imaging; MRI, magnetic resonance imaging; MRS, magnetic resonance spectroscopy; PET, positron emission tomography; and SPECT, single-photon emission computed tomography.

Neuroimaging could provide significant benefits for patients presenting with drug-induced parkinsonism, mainly in those individuals with similar and undifferentiated clinical manifestations to Parkinson’s disease. Neuroimaging techniques have different parameters for assessing brain regions’ structure, function, metabolism, and receptor sites (Figure 1). This literature review describes neuroimaging studies to distinguish Parkinson’s disease from drug-induced parkinsonism.

## 2. Dopamine Radiotracers

### 2.1. History

Several radiotracers were studied in the 1980s. The researchers’ major problems in designing these radioligands were that the agents had an unreliable regional distribution, lower affinity to specific proteins, or slow and long-continued accumulation [10]. The binding of the first radiotracers was relatively non-selective to dopamine, with a similar affinity to other neurotransmitters like noradrenaline and serotonin. Almost a decade later, studies with dopamine transporter (DAT) radiotracers, mainly [123I]-β-CIT, showed significant differences on the valid distribution and affinity to DAT between healthy controls and parkinsonism secondary to 1-methyl-4-phenyl-1,2,3,6-tetrahydropyridine (MTPT) [11].

The cerebellum is usually a reference tissue for assessing the development of new dopamine transporter radiotracers because it does not have these membrane proteins [12]. Among the first radiotracers developed, [123I]-β-CIT has the highest striatal–cerebellum uptake ratio, but the equilibrium for this technique is only achieved after 24 h, which results in delayed scanning. Therefore, researchers developed [123I]-FP-CIT and [123I]-Altropane to shorten the time between intravenous injection and scanning. Nevertheless, these techniques have lower striatal–cerebellum uptake ratios when compared to [123I]-β-CIT [13]. 

The [123I]-FP-CIT Study Group compared the striatal DAT imaging in individuals with probable Parkinson’s disease and patients with essential tremor, in which the sensitivity and specificity were greater than 90%, to differentiate these two pathologies [14]. This study’s results showed the significance of neuroimaging in the assessment of movement disorders, suggesting the inclusion of DAT radiotracers to support the diagnosis of uncertain cases of parkinsonism. Three further pivotal studies evaluated the specificity of DAT imaging for diagnosing Parkinson’s disease. First, the Clinically Uncertain Parkinsonian Syndromes (CUPS) study compared single-time imaging and evaluation [15]. Second, the Query study compared clinical diagnosis to imaging over six months [16]. Third, there was the European multicenter study in which the clinical diagnosis was prospectively compared to imaging over three years [17]. Interestingly, these studies revealed that clinicians’ assessment, compared to imaging, has a high sensitivity and low specificity in diagnosing Parkinson’s disease.

Some years later, 99mTc-TRODAT-1, a metastable technetium-based tropane tracer, was developed. This chemical compound can accumulate faster than other tropane tracers, but it has a low striatal–cerebellum ratio and is not well-extracted by the brain [13]. Therefore, this imaging technique provides a lower accuracy of the subtle dopamine transporter loss, which can result in more false-negative results in individuals with early Parkinson’s disease.

Cocaine analogues were the most studied radiotracers for assessing dopaminergic transport. These analogues are artificially constructed novel chemical compounds from cocaine’s molecular structure. The mechanism of action of cocaine is not yet completely understood. The main characteristic of the purpose for using cocaine analogues as radiotracers is that cocaine can bind tightly at the dopamine transporters blocking the transporter’s function [18]. 

The molecules studied, like cocaine analogues, were attached to several radionuclides. There are four main radionuclides, each with a specific chemical step for including the carried molecules (Table 1) [19]. The properties of the radionuclides are important because they define the protocols of the techniques, such as the time of scanning after contrast infusion and the washing-out period of some medications. It is noteworthy that positron emission tomography (PET) scans are superior to single-photon emission computed tomography (SPECT) for imaging DATs. It is believed that the high energy of positrons provides a higher resolution, resulting in a better image quality with widespread clinical applications [20]. Nevertheless, the majority of the studies in the literature about parkinsonism, including those in patients with drug-induced parkinsonism, performed SPECTs with 123I and 99mTc.

A basic principle should be remembered when assessing dopaminergic radiotracers in parkinsonism. There are significant limitations of the different neuroimaging techniques developed, mainly the dose related to DAT. They can categorize individuals with parkinsonism into degenerative and non-degenerative diseases [21]. In this context, the degenerative diseases may have a similar degenerative pattern as Parkinson’s disease, including corticobasal degeneration, dementia with Lewy bodies, multiple system atrophy parkinsonian type, and progressive supranuclear palsy. On the other hand, the non-degenerative conditions that can be encountered are drug-induced parkinsonism, tremor related to metabolic or functional causes, dystonic tremor, essential tremor, and vascular parkinsonism [22].

### 2.2. Mechanism and General Description of Dopamine Transporter (DAT) Radiotracers

In a healthy individual, dopamine is released in the synaptic cleft, activating post-synaptic neurons. After, dopamine is removed from the synaptic cleft by dopamine transporter (DAT) reuptake. This sodium-dependent DAT is a membrane-spanning protein localized in the presynaptic membrane of neurons. Therefore, DAT imaging measures the functioning of dopaminergic terminals [23].

**Figure 2:**
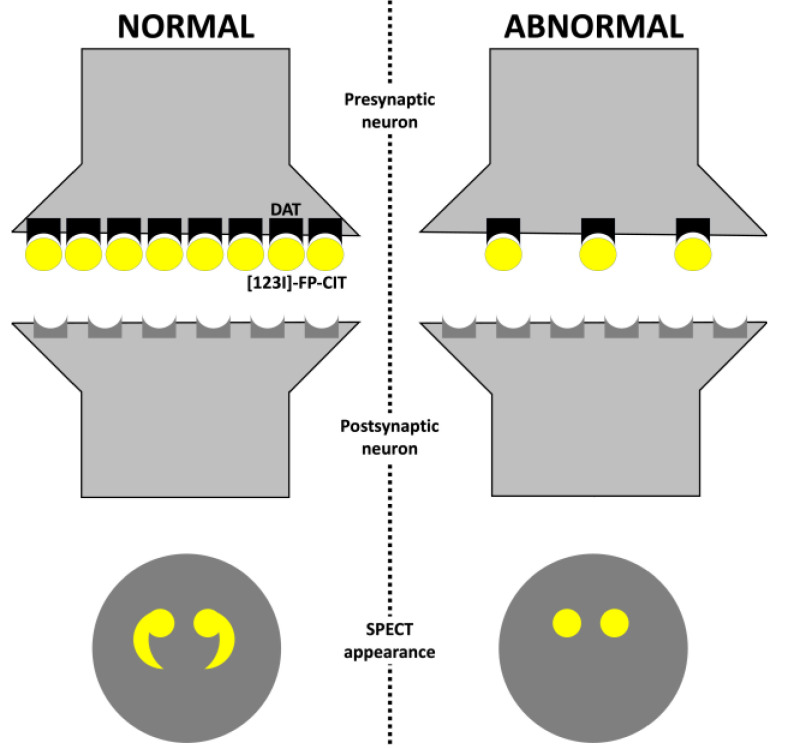
Dopamine transporter radiotracer mechanism. Abbreviations: DAT, dopamine transporter; and SPECT, single-photon emission computed tomography.

In a normal SPECT study, ioflupane (I123) binds to the DATs localized in the presynaptic neurons of the striatum (Figure 2). The SPECT appearance will be a comma- or crescent-shaped activity in the striatum region. The abbreviation for a normal DaTscan is SWEED, which stands for scan without evidence of dopaminergic deficit [24].

In individuals with Parkinson’s disease, neuronal degeneration and loss of dopaminergic neurons can be observed in the striatum. Therefore, only a few DATs will be available in the striatal region for ioflupane to bind. The SPECT appearance will be abnormal with a period- or oval-shaped activity, which can also be reflected in a reduced intensity of one or both sides of the striatal region. In the early stages of Parkinson’s disease, a bilateral reduction in putaminal uptake with a more significant reduction in the putamen contralateral to the most affected limbs with normal uptake in the caudate region is observed [25]. The abbreviation for an abnormal DaTscan is SWIDD, which stands for scans with ipsilateral dopaminergic deficit. Some authors proposed a classification for SPECT imaging appearance of DAT based on the tracing uptake in the basal ganglia region (Table 2) [26,27].

Besides DAT, other mechanisms of the radioligands are related to dihydroxyphenylalanine (DOPA) decarboxylase and vesicular monoamine transporter type 2 (VMAT2). DOPA decarboxylase is a presynaptic radioligand associated with dopamine synthesis. On the other hand, the VMAT2 radioligand is another type of marker of dopaminergic terminals [28].

Some medications may alter tracer binding components due to their affinity to DAT or influencing the metabolism of radioligands or radionuclides. Thus, if possible, stopping them for at least five half-lives before the procedure is advised (Table 3). It is worth mentioning that there are only three absolute contraindications to dopamine transporter imaging: pregnancy, inability to co-operate with the procedure, and hypersensitivity to any component.

We reviewed the Pubmed database to find articles on radiotracers and drug-induced parkinsonism in humans. Table 4 summarizes the radioligands encountered in the literature (Table 4). The authors did not find studies of drug-induced parkinsonism with some radiotracers, such as [11C]-DOPA, [11C]-Raclopride, [18F]-Dihydrotetrabenazine, [123I]-Altropane, [11C]-Altropane, [11C]-WIN35428, [18F]-CFT, [11C]-PE2I, and [123I]-IPT (Table 5).

### 2.3. [123I]-FP-CIT

[123I]-FP-CIT is one of the neuroimaging radiopharmaceutical drugs already approved by the US Food and Drug Administration and the European Medicines Agency (Table 6). [123I]-FP-CIT was first approved in Europe ten years before approval by the FDA. In this context, this radiotracer is the most studied form of DAT imaging in individuals with drug-induced parkinsonism.

There is a significant concern about the influence of aging on dopamine transporter neuroimaging. Autopsy studies revealed a significant reduction in neuromelanin-pigmented neurons in the substantia nigra and the concentration of striatal dopamine transporters with aging [48]. Thus, two large studies were performed to assess the influence of aging on dopamine transporter imaging with healthy subjects [49,50]. The aging effect found on dopamine transporters was independent of race. Moreover, aging did not affect the caudate-to-putamen ratio of DAT binding. Interestingly, some [123I]-FP-CIT studies reported higher striatal binding ratios in the female sex than in males. This effect was strongly associated with age, in which younger individuals had a more pronounced binding effect [51]. Therefore, the aging effect should not affect the diagnosis of parkinsonism secondary to drugs based on the putamen/caudate ratio found in DAT imaging.

Brigo et al. performed a meta-analysis to investigate the significance of neuroimaging to differentiate degenerative from non-degenerative parkinsonism. They found that [123I]-FP-CIT had a sensitivity and specificity of 85 and 80 percent in differentiating idiopathic PD from secondary parkinsonism associated with drugs and vascular conditions [52]. It is noteworthy that there are several limitations in assessing nuclear imaging with meta-analysis. The most significant are related to imaging processing and radiotracer differences, in which every set has a specific protocol and machine.

Table 7 describes the studies performed with [123I]-FP-CIT to differentiate individuals with Parkinson’s disease from parkinsonism secondary to drugs (Table 7).

### 2.4. [123I]-β-CIT

123I-β-CIT is another cocaine derivative first synthesized by the chemist John L. Neumeyer [68]. It is approved by the EMA, but not by the US FDA. [123I]-FP-CIT and [123I]-β-CIT demonstrated a reduction in striatal uptake similarly in people with Parkinson’s disease. But the washed-out time from striatal tissue is up to twenty times faster for [123I]-FP-CIT than [123I]-β-CIT [69]. Moreover, the affinity to DATs was higher with [123I]-FP-CIT. The relatively increased affinity is probably associated with a faster rate of striatal washout and the establishment of transient equilibrium binding conditions at the DAT [70]. Therefore, [123I]-FP-CIT has faster kinetic properties and a relatively high affinity to DAT compared to [123I]-β-CIT.

Eerola et al. investigated the clinical role of [123I]-β-CIT in 195 individuals with movement disorders, of which 12 were diagnosed with drug-induced parkinsonism. The authors found lower [123I]-β-CIT ratios in the putamen, caudate nucleus, and whole striatum in patients with Parkinson’s disease compared to drug-induced parkinsonism. It is noteworthy that [123I]-β-CIT uptake correlated negatively with age (r = −0.39, *p* < 0.01) in parkinsonism secondary to drugs compared to Parkinson’s disease group [71].

Easterford et al. studied three patients that developed valproate-induced parkinsonism. The authors found no abnormality in the [123I]-β-CIT SPECT [72]. In another study, subjects with schizophrenia and long-term antipsychotic use did not present a difference in striatal dopamine transporter density compared to healthy subjects [73]. It is worth mentioning that these studies demonstrated a non-relationship between the dysfunction of striatal neurons and the development of abnormal movements with high doses or long-term use of offending agents.

### 2.5. [99mTc]-TRODAT-1

Technetium-99m is a tropane derivative, [99mTc]-TRODAT-1, which binds to the dopamine transporters. Compared to other dopamine transporter techniques, [99mTc]-TRODAT-1 has several advantages, such as the wide availability of Technetium-99m, lower cost, optimal energy for high-quality imaging, and prompt image visualization due to faster pharmacokinetics [74]. In individuals with Parkinson’s disease, compared to healthy individuals, there is a significant decrease in striatal uptake. It is noteworthy that there is no difference in the 99mTc uptake between early and late-onset Parkinson’s disease, so no correlation with disease progression can be assumed [75].

Fallahi et al. studied [99mTc]-TRODAT-1 in Parkinson’s disease and patients with essential tremor and parkinsonism secondary to drugs. They observed that subjects with non-degenerative forms of parkinsonism show significantly higher normalized basal ganglia uptake than individuals with Parkinson’s disease. The sensitivity and specificity of the technique to differentiate between parkinsonism secondary to drugs and Parkinson’s disease were 80% and 83.3%, respectively [76].

Fabiani et al. assessed 153 individuals with parkinsonism, in which [99mTc]-TRODAT-1 was performed. The authors found that, in the group of parkinsonism secondary to drugs, younger subjects showed the most significant reductions in radiotracer uptake. Moreover, the severity of nonmotor signs was associated with the uptake of the radiotracer. The accuracy, sensitivity, and specificity of [99mTc]-TRODAT-1 to differentiate Parkinson’s disease from drug-induced parkinsonism were 66.9%, 73.1%, and 44%, respectively. It is worth mentioning that these findings with an area under the curve of 0.767 should be highlighted because they are among the lowest already described for imaging methods to differentiate drug-induced from idiopathic Parkinson’s disease [77].

### 2.6. [18F]-DOPA

[18F]-DOPA measures the structural and biochemical integrity of dopaminergic neurons (Table 8). There is a limited number of studies with this radiopharmaceutical drug about drug-induced parkinsonism. A study assessed the efficacy of 18F-DOPA in 13 individuals with severe drug-induced parkinsonism. Putaminal uptake was normal in almost all participants and was predictive of improved clinical signs of parkinsonism. In patients with a reduced uptake, 75% had a progression to clinical parkinsonian symptoms despite the withdrawal of the dopamine receptor antagonist [78]. It is noteworthy that there are data in the literature suggesting that the chronic course of dopamine receptor antagonists may result in a compensatory increase of presynaptic dopamine metabolism, which may contradict the results of Burn et al. [79].

### 2.7. [18F]-AV-133

VMAT2 is responsible for storing monoamines, including dopamine, in presynaptic vesicles located in nerve endings, cell bodies, and dendrites. The reduction of this protein in the striatum reflects the loss of nigrostriatal terminals, in which the measurement of VMAT2 density has been studied to support the potential diagnosis of Parkinson’s disease [80]. Alexander et al. studied the influence of [18F]-AV-133 imaging in the changing clinical management of subjects with clinically uncertain parkinsonian syndrome. The authors found that three individuals with a previous DIP diagnosis had a post-scan DIP diagnosis. However, one individual with a previous neurodegenerative disorder diagnosis had a DIP diagnosis after the scan [81].

### 2.8. [18F]-FP-CIT

FP-CIT has been extensively studied with 123I radionuclide, but few studies with 18F exist. The attachment of 18F radionuclide to the FP-CIT ligand provides a radiotracer with high signal-to-noise ratios with low artifacts and fast kinetics. Hong et al. assessed 50 individuals with drug-induced parkinsonism, in which the participants were divided into full and partial recovery. Relative lower ligand uptake was observed in the ventral striatum, anterior putamen, and posterior putamen in the patients that achieved partial recovery compared to the complete recovery group [82].

Oh et al. used [18F]-FP-CIT and imaging software to discover the standardized uptake value ratios between patients with parkinsonism secondary to drugs and healthy individuals [83]. The analyzed volume-of-interest template revealed a decreased monoamine availability in the thalamus in individuals with drug-induced parkinsonism compared to healthy individuals. Interestingly, they did not find any difference in the concentrations of monoamines in other subregions (putamen, globus pallidus, and ventral striatum).

Shin et al. studied 76 patients with DIP that [18F]-FP-CIT was performed. The authors found that symmetric parkinsonism was more prevalent. Moreover, the duration of drug exposure before the onset of parkinsonism was shorter in the patients with normal imaging than those with abnormal imaging [84].

### 2.9. Neuroimaging and Receptors Occupancy

Some radiotracers related to dopaminergic metabolism are used to assess dopamine receptors’ occupancy by neuroleptic agents in humans. PET studies have shown that D2 receptor occupancy at above eighty percent invariably results in extrapyramidal side effects including, but not limited to, parkinsonian motor symptoms [85]. It is noteworthy that the knowledge of this association can lead to the development of medications with lower or specific dopaminergic occupancy, leading to fewer motor side effects.

Olanzapine was one of the medications whose doses were further adjusted with DAT imaging. Comparing olanzapine 5 mg and 20 mg a day showed an occupancy of 60 and 83% of the dopaminergic receptors, respectively, without differences in clinical efficacy at these doses [86]. Another medication that was already tested with dopamine receptors occupancy is risperidone. In this context, risperidone 4 mg showed an occupancy varying from 70% to 80% [87].

The graphical description of the dopamine receptors’ occupancy can be represented by hyperbole. In this way, increases in higher doses of dopamine blockers will not be beneficial because the patient will achieve an effective plateau [88]. Therefore, several studies with DAT imaging with lower doses of antipsychotics already showed a similar efficacy to higher antipsychotic doses with significantly fewer side effects.

## 3. [18F]-Fluorodeoxyglucose ([18F]-FDG) PET

[18F]-FDG is a radiotracer that can mark the tissue uptake of glucose, which is closely correlated with some metabolism pathways. Several studies already evaluated the use of [18F]-FDG in supporting the diagnosis of Parkinson’s disease, in which the specific pattern encountered is an increased uptake of the striatum, thalamus, motor cortex, and cerebellum. On the other hand, the temporoparietooccipital cortex is believed to have a lower uptake [89].

Kotomin et al. studied the metabolic brain imaging approach using the 18F-FDG PET and spatial covariance analysis to find possible factors that could predict drug-induced parkinsonism. They found that the expression of a Parkinson’s-disease-related pattern on 18F-FDG was commonly related to the development of parkinsonism secondary to drugs. However, this pattern was also observed in patients receiving antipsychotics without motor symptoms [90].

## 4. [123I]-MIBG Cardiac Imaging

[123I]-MIBG scintigraphy assesses the integrity of the cardiac sympathetic nerve terminals. Studies showed that this neuroimaging technique can be used to differentiate Parkinson’s disease from other forms of parkinsonism [91]. A limited number of studies assessing [123I]-MIBG scintigraphy in parkinsonism secondary to drugs have already been published in the literature.

Lee et al. evaluated 52 individuals with parkinsonism, of which 20 were diagnosed with drug-induced parkinsonism. Ten percent of the subjects with a drug-induced parkinsonism diagnosis showed a reduced uptake compared to patients with Parkinson’s disease. The two individuals with drug-induced parkinsonism and a reduced uptake also had no improvement in their motor symptoms with drug discontinuation. However, both patients significantly improved motor symptoms with the levodopa trial [92].

Lee et al. performed a second study with cross-cultural smell identification (CCSI) testing in 54 individuals with parkinsonism, of which 15 were diagnosed with drug-induced parkinsonism. One of the participants had low CCSI scores and a reduced uptake of [123I]-MIBG, which can suggest that olfactory tests may help distinguish between parkinsonism secondary to drugs and subclinical Parkinson’s disease. It is noteworthy that the CCSI test can be performed quickly in the outpatient clinic and is inexpensive compared to scintigraphy [93].

Kim et al. studied the combination of [123I]-MIBG and [123I]-FP-CIT SPECT in 36 individuals with parkinsonism, of which 20 had a diagnosis of drug-induced parkinsonism. In this study, 80% of the individuals with drug-induced parkinsonism had normal cardiac imaging and DAT imaging studies. Interestingly, two individuals presented with normal [123I]-FP-CIT and decreased [123I]-MIBG uptakes. After two years, these individuals had worsened parkinsonian symptoms. A second imaging sequence showed a reduced uptake of [123I]-FP-CIT and [123I]-MIBG. Therefore, these findings suggest cardiac abnormalities are found before striatal region lesions. In this way, it is possible that those patients with probable drug-induced parkinsonism and normal DAT scans with less improvement after drug discontinuation will benefit significantly from cardiac imaging [94].

Shafie et al. studied 44 patients with parkinsonism secondary to drugs and 32 patients with idiopathic Parkinson’s disease. The authors found that the difference [123I]-MIBG uptake between the Parkinson’s disease and drug-induced parkinsonism groups was significant. Moreover, Shafie et al. reported that [123I]-MIBG scans could be used to determine the prognosis of people with parkinsonism secondary to drugs. The subjects with drug-induced parkinsonism that did not improve motor symptoms after offending drug discontinuation had a low heart-to-mediastinum ratio [95].

## 5. Magnetic Resonance Imaging (MRI)—The Swallowtail Appearance

Nigrosomes are small clusters of dopaminergic cells within the healthy substantia nigra. They can have a hypersignal in the axial section, with either a linear or comma appearance. They are bordered anteriorly, laterally, and medially by a low-intensity signal, giving it a swallow-tailed appearance. A loss of the normal swallowtail appearance of the susceptibility signal pattern in the substantia nigra on axial imaging is one of the diagnostic signs for Parkinson’s disease [96].

Sung et al. studied 20 individuals with drug-induced parkinsonism and 29 with Parkinson’s disease. The individuals were first assessed with [18F]-FP-CIT imaging after nigrosome-1 3T imaging was evaluated. Then, 85% of the patients with parkinsonism secondary to drugs were interpreted as normal 3T imaging findings, in which the sensitivity was 100%, specificity 85%, and accuracy 93.9% [97].

Studies with an ultra-high-field MRI (7T) showed significant sensitivity and specificity for diagnosing Parkinson’s disease based on the loss of the swallowtail appearance [98]. Therefore, future investigations with high-quality neuroimaging could be a promising field for supporting the diagnosis of non-degenerative causes of parkinsonism, such as parkinsonism secondary to drugs.

## 6. Transcranial Ultrasound

B-mode transcranial ultrasonography was already studied to support the diagnosis of Parkinson’s disease. This imaging method, when compared to other techniques, has significant advantages, such as relatively low costs, broad availability, and a noninvasive approach. The characteristic finding in patients with Parkinson’s disease is an increased echogenicity of the mesencephalic substantia nigra region, which is probably related to iron deposition [99]. The presence of this sign is highly specific to the diagnosis of a degenerative form of parkinsonism. Nevertheless, the sensitivity depends on the exact cut-off value of the substantia nigra area used and the type of ultrasound machine [100].

Bouwmans et al. assessed 196 individuals with parkinsonism of unclear etiology. After two years of follow-up, seven individuals were diagnosed with drug-induced parkinsonism. All the individuals were evaluated with [123I]-FP-CIT and B-mode transcranial ultrasonography. Ultrasonography accurately identified drug-induced parkinsonism in 86% of the subjects [101].

Olivares Romero et al.’s study enrolled 20 subjects diagnosed with possible drug-induced parkinsonism in which the offending agent was discontinued. The authors found a sensitivity of 80% and a negative predictive value of 87.5% with the evaluation of echogenicity in the substantia nigra and the lentiform nucleus regions [102].

Oh et al. studied the significance of early transcranial ultrasound in diagnosing drug-induced parkinsonism. They found pure drug-induced parkinsonism has different echogenicity patterns than unmasked Parkinson’s disease. The substantia nigra hyperechogenicity in patients with unmasked Parkinson’s disease showed a sensitivity of 75% and a specificity of 91.1%. Therefore, early transcranial ultrasonography findings may be useful in predicting unmasked Parkinson’s disease in individuals presenting with possible parkinsonism secondary to drugs [103].

## 7. Optical Coherence Tomography

Patients with Parkinson’s disease commonly present visual symptoms, especially perceptual disturbances such as impairment in stereopsis, visual illusions, and visual hallucinations. Patients with Parkinson’s disease have a decreased average capillary retinal nerve fiber layer in every quadrant [104]. Moreover, Jimenez et al. proposed an equation to determine the Parkinson’s disease progression based on the Unified Parkinson’s Disease Rating Scale (UPDRS) total score and the retinal nerve fiber layer thickness measured by optical coherence tomography [105].

Suh et al. assessed 97 individuals with Parkinson’s disease and 27 with parkinsonism secondary to drugs using optical coherence tomography and [18F] N-(3-fluoropropyl)-2b-carbon ethoxy-3b-(4-iodophenyl) nortropane (FP-CIT). They compared the two groups’ peripapillary retinal nerve fiber layer and macular retinal thickness. There were no significant differences in peripapillary and macular retinal thickness values [106]. Suh et al.’s study is important because it showed that, in the early stages of drug-induced parkinsonism, there is no benefit in measuring these optic parameters to differ from early Parkinson’s disease.

## 8. Expert Recommendations

The time between “drug discontinuation” and “neuroimaging” in drug-induced parkinsonism is one of the main concerns in clinical practice (Table 9). Some authors state that neuroimaging should be carried out within the first month of the drug withdrawal. Others propose that neuroimaging should only be requested for patients with partial or no improvement of motor symptoms after six months of discontinuing the offending drug.

The best approach may be to search for systematic studies in the literature regarding specific drugs or classes of drugs and their association with parkinsonism, for example, an individual presenting with parkinsonism after flunarizine therapy. Rissardo et al. described a systematic review of cinnarizine and flunarizine associated with movement disorders [107]. The authors found that 94.85% of the individuals who developed cinnarizine- or flunarizine-induced parkinsonism had a complete recovery within six months of drug discontinuation. Therefore, if the individual with parkinsonism does not show improvement within the first six months of drug discontinuation, neuroimaging should be requested to evaluate the dopaminergic pathway in the striatal region.

**Figure 3:**
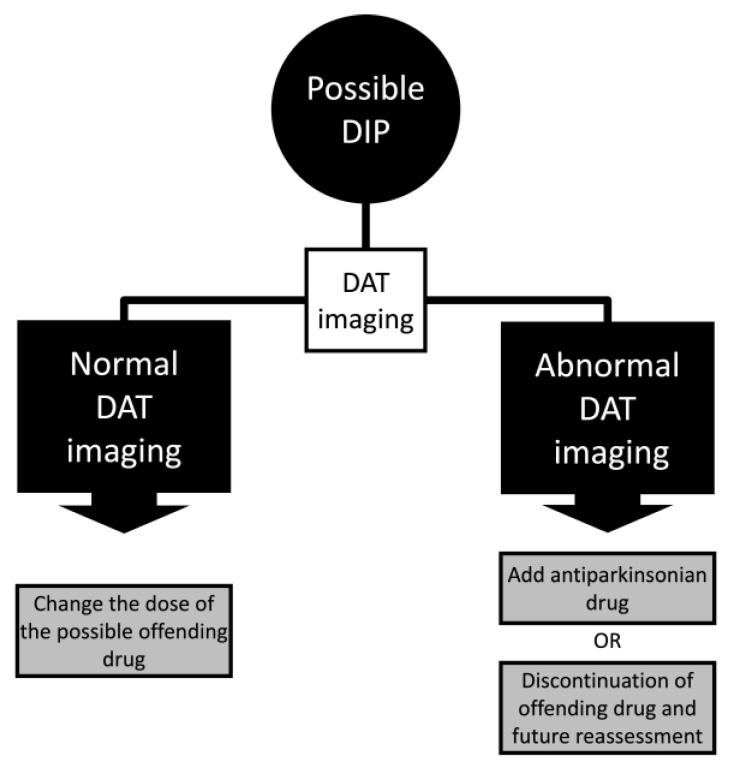
Algorithm of drug-induced parkinsonism (DIP) management with dopamine transporter (DAT) imaging.

Another important clue for using DAT imaging is that in cases where the drug possibly causing abnormal motor symptoms cannot be discontinued. One possible approach could be to use DAT imaging to titrate the offending drug or include antiparkinsonian drugs in the patient’s therapy (Figure 3). Moreover, the offending drug discontinuation with a further reassessment in patients with abnormal DAT imaging and a low probability of developing Parkinson’s disease should be carried out. One possible explanation in these cases is the subclinical drug-exacerbated parkinsonism, in which the individual develops parkinsonism with a symmetrical abnormality in DAT and neurologic examination after a short term of the medication, mainly dopamine-blocking agents.

There are several limitations in the present study. First, most studies included did not specifically assess only parkinsonism secondary to drugs. In their analysis, the authors included patients with drug-induced parkinsonism with vascular parkinsonism and essential tremor to provide larger samples. Second, there is a significantly low number of studies evaluating the use of neuroimaging techniques to distinguish between neurodegenerative and drug-induced parkinsonism. It is noteworthy that many studies were performed by the same groups, which could lead to the repetition of selective bias. Third, a systematic literature search was only performed to describe the radiotracers.

## 9. Future Studies

The literature on neuroimaging in drug-induced movement disorders is scarce. There are only a few studies assessing different types of radiotracers. After these initial studies, head-to-head trials comparing the different radiotracers are needed to evaluate sensitivity and specificity. Future studies should develop protocols for performing neuroimaging in individuals with drug-induced parkinsonism. The best time to request neuroimaging and the influence of different medications with washing-out periods should be studied.

Clinical symptoms associated with invasive and non-invasive neuroimaging methods must be studied. These results can provide a significant change in clinical practice, leading to the development of calculators and scores that may help the clinician to provide prognosis and specific management. Another approach for neuroimaging in drug-induced parkinsonism is the dual imaging algorithm, which involves performing imaging in the central and peripheral nervous system. This algorithm has been used in the last decade to differentiate Parkinson’s disease from other forms of parkinsonism. It is worth mentioning that individuals with drug-induced parkinsonism will present normal results in both studies.

## 10. Conclusions

The neuroimaging techniques already studied with drug-induced parkinsonism are dopaminergic radiotracers, [18F]-FDG, [123I]-MIBG cardiac scintigraphy, structural MRI, transcranial ultrasound, and optical coherence tomography. The procedure with the highest number of publications is dopamine transporter imaging. [123I]-FP-CIT is the most frequently performed and studied imaging agent with drug-induced parkinsonism. There are significant differences between the radiotracers, which future studies should assess. Neuroimaging in drug-induced parkinsonism might improve diagnosis, prognosis, and appropriate medication use, translating into better patient care with favorable outcomes.

## Figures and Tables

**Table 1:** Properties of selected radionuclides.

Radionuclide	Half-Life	Type of Emission	Energy	Intensity
11C	20.3 min	Positron	961 Kev	99.8%
18F	109.7 min	Positron	634 Kev	97%
123I	13.3 h	Gamma	159 Kev	83%
99mTc	6 h	Gamma	140 Kev	89%

**Table 2:** Dopamine transporter uptake SPECT appearance classification.

Grade		Description	Diagram
Normal		Caudate nucleus appears like a “full stop” and putamen-like “tail” (whole appearance is like a comma on both sides)	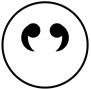
SWIDD (scans with ipsilateral dopaminergic deficit)	Type 1	Normal “full stop” with unilateral disappearing “comma” (asymmetrical loss of putaminal tail)	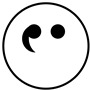 OR 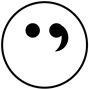
	Type 2	“Two full stops” (bilateral loss of putaminal tails)	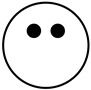
	Type 3	“Disappearing full stops” (partial to complete loss of caudate and putaminal signals)	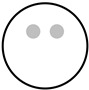

**Table 3:** Recommendations regarding the influence of different agents in dopamine transporter imaging.

Agent		Agent Effect in the Striatal Binding	Half-Life	Recommended Time to Hold before Imaging	Reference
Adrenergic agonists	Phenylephrine	Increased when infused at high doses	NA	NA	[29]
	Norepinephrine		NA	NA	
Amphetamines		Decreased	5–30 h	3–7 days	[30]
Anesthetics	Ketamine	Decreased	3 h	15 h	[31]
	Phencyclidine		7–46 h	10 days	
	Isoflurane		2.1 min	10.5 min	
Anticholinergics	Benztropine	Decreased	12–24 h	5 days	[32]
	Scopolamine-like	Increased, but no effect on visual assessment	9.5 h	2 days	[33]
Antidepressants	Bupropion	Decreased/none	12–30 h	8 days	[34]
	Mazindol	Decreased	10–13 h	3 days	[29]
	Radafaxine	Decreased	NA	NA	[35]
	SNRIs	Increased, but no effect on visual assessment	NA	Not required	[36]
	SSRIs	Increased, but no effect on visual assessment	NA	Not required	[37]
	Tricyclic antidepressants	None	NA	Not required	[29]
Antiparkinsonian medication	L-DOPA	None, but possible downregulation of dopamine transporters	NA	Not required	[38]
	Dopamine agonists		NA	Not required	[39]
	NMDA receptors blockers		NA	Not required	[29]
	MAO-B inhibitors		NA	Not required	[40]
	COMT inhibitors		NA	Not required	[29]
Cholinesterase inhibitors		None	NA	Not required	[41]
CNS stimulants	Phentermine	Decreased	25 h	6 days	[29]
	Ephedrine		6 h	30 h	
Cocaine		Decreased	1 h	2 days	[42]
Estrogen replacement post menopause		Increased, but no effect on visual assessment	NA	Not required	[29]
Lithium		Decreased	24 h	5 days	[21]
Menstrual cycle		None	NA	NA	[43]
Modafinil		Decreased	15 h	3 days	[44]
Neuroleptics/antipsychotics		None	NA	Not required	[45]
Opioids	Fentanyl	Decreased	2–4 h	20 h	[46]
	Naltrexone	None	NA	Not required	[47]

Abbreviations: CNS, central nervous system; COMT, catechol-O-methyltransferase; MAO-B, monoamine oxidase B; NA, not applicable/not available; NMDA, N-methyl-D-aspartate; SNRIs, serotonin–norepinephrine reuptake inhibitor; and SSRIs, selective serotonin reuptake inhibitor.

**Table 4:** Radiotracers and drug-induced parkinsonism.

Abbreviation	Other Names	Approval	PET/SPECT	Radiopharmaceutical Agent	Mechanism
[123I]-FP-CIT	DaTscan, Ioflupane	FDA EMA	SPECT	123I-N-3-fluoropropyl-2bcarboxymethoxy- 3b-(4-iodophenyl) tropane	Pre-synaptic. DAT.
[123I]-β-CIT	Dopascan	EMA	SPECT	123I-(2)-2β-carboxymethoxy-3b-(4-iodophenyl) tropane	Pre-synaptic. DAT.
[99mTc]-TRODAT-1	NA	NA	SPECT	99mTc-TRODAT-1 ([2-[2-[3-(4-chlorophenyl)-8-methyl-8-azabicyclo [3,2,1]oct-2-yl] methyl](2-mercaptoethyl) -amino] ethyl] amino] ethanethiolato (3-)-N2, N2′, S2, S2′]oxo-[1R-(exo-exo)])	Pre-synaptic. DAT.
[18F]-DOPA	Fluorodopa	FDA EMA	PET	6-[18F]fluoro-L-3,4-dihydroxyphenylalanine	Pre-synaptic. DOPA decarboxylase.
[18F]-AV-133	NA	NA	PET	9-[(18)F]fluoropropyl-(+)-dihydrotetrabenazine	Pre-synaptic. VMAT2.
[18F]-FP-CIT	NA	NA	PET	18F-N-(3-fluoropropyl)-2beta-carbon ethoxy-3beta-(4-iodophenyl) nortropane	Pre-synaptic. DAT.

Abbreviations: DAT, dopamine transporter; DOPA, dihydroxyphenylalanine; EMA, European Medicines Agency; FDA, US Food and Drug Administration; NA, not available/not applicable; PET, positron emission tomography; SPECT, single-photon emission computed tomography; and VMAT2, vesicular monoamine transporter type 2.

**Table 5:** FreeText and MeSH search terms in the US National Library of Medicine.

Query	MeSH Terms	Results
([123I]-FP-CIT) AND (drug-induced parkinsonism)	(“2 carbomethoxy 8 3 fluoropropyl 3 4 iodophenyl tropane”[Supplementary Concept] OR “2 carbomethoxy 8 3 fluoropropyl 3 4 iodophenyl tropane”[All Fields] OR “123i fp cit”[All Fields]) AND (“drug-induced”[All Fields] AND (“parkinson disease”[MeSH Terms] OR (“parkinson”[All Fields] AND “disease”[All Fields]) OR “parkinson disease”[All Fields] OR “parkinsons”[All Fields] OR “parkinson”[All Fields] OR “parkinson s”[All Fields] OR “parkinsonian disorders”[MeSH Terms] OR (“parkinsonian”[All Fields] AND “disorders”[All Fields]) OR “parkinsonian disorders”[All Fields] OR “parkinsonism”[All Fields] OR “parkinsonisms”[All Fields] OR “parkinsons s”[All Fields]))	33
([18F]-FP-CIT) AND (drug-induced parkinsonism)	(“2 carbomethoxy 8 3 fluoropropyl 3 4 iodophenyl tropane”[Supplementary Concept] OR “2 carbomethoxy 8 3 fluoropropyl 3 4 iodophenyl tropane”[All Fields] OR “18f fp cit”[All Fields]) AND (“drug-induced”[All Fields] AND (“parkinson disease”[MeSH Terms] OR (“parkinson”[All Fields] AND “disease”[All Fields]) OR “parkinson disease”[All Fields] OR “parkinsons”[All Fields] OR “parkinson”[All Fields] OR “parkinson s”[All Fields] OR “parkinsonian disorders”[MeSH Terms] OR (“parkinsonian”[All Fields] AND “disorders”[All Fields]) OR “parkinsonian disorders”[All Fields] OR “parkinsonism”[All Fields] OR “parkinsonisms”[All Fields] OR “parkinsons s”[All Fields]))	30
([18F]-DOPA) AND (drug-induced parkinsonism)	(“fluorodopa f 18”[Supplementary Concept] OR “fluorodopa f 18”[All Fields] OR “18f dopa”[All Fields]) AND (“drug-induced”[All Fields] AND (“parkinson disease”[MeSH Terms] OR (“parkinson”[All Fields] AND “disease”[All Fields]) OR “parkinson disease”[All Fields] OR “parkinsons”[All Fields] OR “parkinson”[All Fields] OR “parkinson s”[All Fields] OR “parkinsonian disorders”[MeSH Terms] OR (“parkinsonian”[All Fields] AND “disorders”[All Fields]) OR “parkinsonian disorders”[All Fields] OR “parkinsonism”[All Fields] OR “parkinsonisms”[All Fields] OR “parkinsons s”[All Fields]))	9
([123I]-β-CIT) AND (drug-induced parkinsonism)	“123i beta cit”[All Fields] AND (“drug-induced”[All Fields] AND (“parkinson disease”[MeSH Terms] OR (“parkinson”[All Fields] AND “disease”[All Fields]) OR “parkinson disease”[All Fields] OR “parkinsons”[All Fields] OR “parkinson”[All Fields] OR “parkinson s”[All Fields] OR “parkinsonian disorders”[MeSH Terms] OR (“parkinsonian”[All Fields] AND “disorders”[All Fields]) OR “parkinsonian disorders”[All Fields] OR “parkinsonism”[All Fields] OR “parkinsonisms”[All Fields] OR “parkinsons s”[All Fields]))	4
([99mTc]-TRODAT-1) AND (drug-induced parkinsonism)	(“technetium tc 99m trodat 1”[Supplementary Concept] OR “technetium tc 99m trodat 1”[All Fields] OR “99mtc trodat 1”[All Fields]) AND (“drug-induced”[All Fields] AND (“parkinson disease”[MeSH Terms] OR (“parkinson”[All Fields] AND “disease”[All Fields]) OR “parkinson disease”[All Fields] OR “parkinsons”[All Fields] OR “parkinson”[All Fields] OR “parkinson s”[All Fields] OR “parkinsonian disorders”[MeSH Terms] OR (“parkinsonian”[All Fields] AND “disorders”[All Fields]) OR “parkinsonian disorders”[All Fields] OR “parkinsonism”[All Fields] OR “parkinsonisms”[All Fields] OR “parkinsons s”[All Fields]))	4
([18F]-AV-133) AND (drug-induced parkinsonism)	(“florbenazine f 18”[Supplementary Concept] OR “florbenazine f 18”[All Fields] OR “18f av 133”[All Fields]) AND (“drug-induced”[All Fields] AND (“parkinson disease”[MeSH Terms] OR (“parkinson”[All Fields] AND “disease”[All Fields]) OR “parkinson disease”[All Fields] OR “parkinsons”[All Fields] OR “parkinson”[All Fields] OR “parkinson s”[All Fields] OR “parkinsonian disorders”[MeSH Terms] OR (“parkinsonian”[All Fields] AND “disorders”[All Fields]) OR “parkinsonian disorders”[All Fields] OR “parkinsonism”[All Fields] OR “parkinsonisms”[All Fields] OR “parkinsons s”[All Fields]))	1
([123I]-IPT) AND (drug-induced parkinsonism)	(“n 3 iodopropen 1 yl 2 carbomethoxy 3 4 chlorophenyl tropane”[Supplementary Concept] OR “n 3 iodopropen 1 yl 2 carbomethoxy 3 4 chlorophenyl tropane”[All Fields] OR “123i ipt”[All Fields]) AND (“drug-induced”[All Fields] AND (“parkinson disease”[MeSH Terms] OR (“parkinson”[All Fields] AND “disease”[All Fields]) OR “parkinson disease”[All Fields] OR “parkinsons”[All Fields] OR “parkinson”[All Fields] OR “parkinson s”[All Fields] OR “parkinsonian disorders”[MeSH Terms] OR (“parkinsonian”[All Fields] AND “disorders”[All Fields]) OR “parkinsonian disorders”[All Fields] OR “parkinsonism”[All Fields] OR “parkinsonisms”[All Fields] OR “parkinsons s”[All Fields]))	0
([11C]-DOPA) AND (drug-induced parkinsonism)	“11c dopa”[All Fields] AND (“drug-induced”[All Fields] AND (“parkinson disease”[MeSH Terms] OR (“parkinson”[All Fields] AND “disease”[All Fields]) OR “parkinson disease”[All Fields] OR “parkinsons”[All Fields] OR “parkinson”[All Fields] OR “parkinson s”[All Fields] OR “parkinsonian disorders”[MeSH Terms] OR (“parkinsonian”[All Fields] AND “disorders”[All Fields]) OR “parkinsonian disorders”[All Fields] OR “parkinsonism”[All Fields] OR “parkinsonisms”[All Fields] OR “parkinsons s”[All Fields]))	0
([11C]-DOPA) AND (drug-induced parkinsonism)	“11c dopa”[All Fields] AND (“drug-induced”[All Fields] AND (“parkinson disease”[MeSH Terms] OR (“parkinson”[All Fields] AND “disease”[All Fields]) OR “parkinson disease”[All Fields] OR “parkinsons”[All Fields] OR “parkinson”[All Fields] OR “parkinson s”[All Fields] OR “parkinsonian disorders”[MeSH Terms] OR (“parkinsonian”[All Fields] AND “disorders”[All Fields]) OR “parkinsonian disorders”[All Fields] OR “parkinsonism”[All Fields] OR “parkinsonisms”[All Fields] OR “parkinsons s”[All Fields]))	0
[18F]-Dihydrotetrabenazine	“18f dihydrotetrabenazine”[All Fields] AND (“drug-induced”[All Fields] AND (“parkinson disease”[MeSH Terms] OR (“parkinson”[All Fields] AND “disease”[All Fields]) OR “parkinson disease”[All Fields] OR “parkinsons”[All Fields] OR “parkinson”[All Fields] OR “parkinson s”[All Fields] OR “parkinsonian disorders”[MeSH Terms] OR (“parkinsonian”[All Fields] AND “disorders”[All Fields]) OR “parkinsonian disorders”[All Fields] OR “parkinsonism”[All Fields] OR “parkinsonisms”[All Fields] OR “parkinsons s”[All Fields]))	0
([123I]-Altropane) AND (drug-induced parkinsonism)	“123i altropane”[All Fields] AND (“drug-induced”[All Fields] AND (“parkinson disease”[MeSH Terms] OR (“parkinson”[All Fields] AND “disease”[All Fields]) OR “parkinson disease”[All Fields] OR “parkinsons”[All Fields] OR “parkinson”[All Fields] OR “parkinson s”[All Fields] OR “parkinsonian disorders”[MeSH Terms] OR (“parkinsonian”[All Fields] AND “disorders”[All Fields]) OR “parkinsonian disorders”[All Fields] OR “parkinsonism”[All Fields] OR “parkinsonisms”[All Fields] OR “parkinsons s”[All Fields]))	0
([11C]-Altropane) AND (drug-induced parkinsonism)	“11c altropane”[All Fields] AND (“drug-induced”[All Fields] AND (“parkinson disease”[MeSH Terms] OR (“parkinson”[All Fields] AND “disease”[All Fields]) OR “parkinson disease”[All Fields] OR “parkinsons”[All Fields] OR “parkinson”[All Fields] OR “parkinson s”[All Fields] OR “parkinsonian disorders”[MeSH Terms] OR (“parkinsonian”[All Fields] AND “disorders”[All Fields]) OR “parkinsonian disorders”[All Fields] OR “parkinsonism”[All Fields] OR “parkinsonisms”[All Fields] OR “parkinsons s”[All Fields]))	0
([11C]-WIN35428) AND (drug-induced parkinsonism)	“11C”[All Fields] AND “win35428”[All Fields] AND (“drug-induced”[All Fields] AND (“parkinson disease”[MeSH Terms] OR (“parkinson”[All Fields] AND “disease”[All Fields]) OR “parkinson disease”[All Fields] OR “parkinsons”[All Fields] OR “parkinson”[All Fields] OR “parkinson s”[All Fields] OR “parkinsonian disorders”[MeSH Terms] OR (“parkinsonian”[All Fields] AND “disorders”[All Fields]) OR “parkinsonian disorders”[All Fields] OR “parkinsonism”[All Fields] OR “parkinsonisms”[All Fields] OR “parkinsons s”[All Fields]))	0
[18F]-CFT	“18F”[All Fields] AND “cft”[All Fields] AND (“drug-induced”[All Fields] AND (“parkinson disease”[MeSH Terms] OR (“parkinson”[All Fields] AND “disease”[All Fields]) OR “parkinson disease”[All Fields] OR “parkinsons”[All Fields] OR “parkinson”[All Fields] OR “parkinson s”[All Fields] OR “parkinsonian disorders”[MeSH Terms] OR (“parkinsonian”[All Fields] AND “disorders”[All Fields]) OR “parkinsonian disorders”[All Fields] OR “parkinsonism”[All Fields] OR “parkinsonisms”[All Fields] OR “parkinsons s”[All Fields]))	0
([11C]-PE2I) AND (drug-induced parkinsonism)	(“n 3 iodoprop 2 enyl 2 beta carbomethoxy 3 4 methylphenyl nortropane”[Supplementary Concept] OR “n 3 iodoprop 2 enyl 2 beta carbomethoxy 3 4 methylphenyl nortropane”[All Fields] OR “11c pe2i”[All Fields]) AND (“drug-induced”[All Fields] AND (“parkinson disease”[MeSH Terms] OR (“parkinson”[All Fields] AND “disease”[All Fields]) OR “parkinson disease”[All Fields] OR “parkinsons”[All Fields] OR “parkinson”[All Fields] OR “parkinson s”[All Fields] OR “parkinsonian disorders”[MeSH Terms] OR (“parkinsonian”[All Fields] AND “disorders”[All Fields]) OR “parkinsonian disorders”[All Fields] OR “parkinsonism”[All Fields] OR “parkinsonisms”[All Fields] OR “parkinsons s”[All Fields]))	0

**Table 6:** Labeled indications for [123I]-FP-CIT.

Agency	Descriptor
European Medicines Agency	This medicinal product is for diagnostic use only. DaTSCAN is indicated for detecting loss of functional dopaminergic neuron terminals in the striatum:
	(1) In patients with clinically uncertain parkinsonian syndromes, in order to help differentiate essential tremor from parkinsonian syndromes related to idiopathic Parkinson’s disease, multiple system atrophy, and progressive supranuclear palsy. DaTSCAN is unable to discriminate between Parkinson’s disease, multiple system atrophy, and progressive supranuclear palsy.
	(2) To help differentiate probable dementia with Lewy bodies from Alzheimer’s disease. DaTSCAN is unable to discriminate between dementia with Lewy bodies and Parkinson’s disease dementia.
US Food and Drug Administration	DaTscan is a radiopharmaceutical indicated for striatal dopamine transporter visualization using single-photon emission computed tomography brain imaging to assist in the evaluation of adult patients with suspected parkinsonian syndromes. In these patients, DaTscan may be used to help differentiate essential tremor from tremor due to parkinsonian syndromes (idiopathic Parkinson’s disease, multiple system atrophy, and progressive supranuclear palsy). DaTscan is an adjunct to other diagnostic evaluations.

**Table 7:** [123I]-FP-CIT studies of individuals with drug-induced parkinsonism.

Reference	Population	Main Findings
Booij et al. (2001) [53]	8 PD, 3 DIP, 4 VP	All the individuals with DIP presented normal SPECT imaging.
Lorberboym et al. (2006) [54]	20 patients with parkinsonism	The clinical symptoms between patients with normal and abnormal scans were similar. Nine individuals had normal scans and were diagnosed with DIP.
Manoharan et al. (2007) [27]	13 PD, 1 DIP, 1 VP	Two individuals were using neuroleptics and developed parkinsonism. One had abnormal imaging and was managed as Parkinson’s disease; the other had normal imaging and was treated as DIP.
Vlaar et al. (2008) [55]	248 patients with parkinsonism	The mean odds ratio to differentiate PD from DIP was 36 (95% confidence interval, 2–697) with [123I]-FP-CIT. The specificity was 100%, and the sensitivity was 80%.
Tinazzi et al. (2008) [56]	32 DIP, 26 healthy controls	Uptake was normal in 18 patients and reduced in 14 patients, who were diagnosed with PD unmasked by the anti-dopaminergic medication. The symmetry of motor symptoms and orofacial dyskinesias were more frequent in patients with normal uptake.
Tinazzi et al. (2009) [57]	10 DIP with normal SPECT (group 1), 9 DIP with abnormal SPECT (group 2)	Upon follow-up, it was observed that all patients in group 1 had normal SPECT results and their UPDRS motor score values had not progressed. Meanwhile, patients in group 2 showed progression in both putaminal dopaminergic denervation on the scan and UPDRS motor scores. Levodopa treatment had a positive impact on motor symptoms in 30% of patients in group 1 and in 88.9% of patients in group 2.
Diaz-Corrales et al. (2010) [58]	25 PD unmasked by antidopaminergic drugs, 22 PD without a previous antidopaminergic treatment, 32 DIP	Normal results in 29 (90.6%) patients with DIP. Abnormal uptake was observed in all patients with PD (qualitative assessments of SPECT images).
Hambye et al. (2010) [59]	22 individuals with probable amiodarone-induced parkinsonism	The patients were undifferentiated by clinical symptoms. Imaging was used to design specific management. Individuals who had normal imaging were managed with an adjustment of amiodarone dose. Those individuals with abnormal imaging were treated with antiparkinsonian medications.
Cuberas-Borros et al. (2011) [60]	20 PD, 20 DIP, and 20 essential tremor	Decreased uptake in the putamen nuclei was only noted in patients with PD.
Tinazzi et al. (2012) [61]	448 schizophrenic patients treated with antipsychotics for at least 6 months	DIP was found in 33% of patients and 42% of patients with neuroimaging abnormalities had PD unmasked by the drug.
Olivares Romero et al. (2013) [62]	19 DIP evaluated at least 6 months after discontinuation of antidopaminergic drugs	Sensitivity of 66.7%, specificity and positive predictive value of 100%, negative predictive value of 86.7%, and a negative likelihood ratio of 0.33 for diagnosis of iatrogenic parkinsonism or subclinical drug-exacerbated parkinsonism.
Tinazzi et al. (2014) [63]	60 patients with schizophrenia and parkinsonism with SPECT at baseline evaluation (normal SPECT = 33; abnormal SPECT = 27)	Patients with baseline abnormal SPECT had higher UPDRS motor scores at follow-up and were more likely to respond to levodopa trial.
Morley et al. (2017) [64]	33 patients with DIP	The patients were evaluated with imaging and olfactory testing. Imaging was abnormal in 21% cases. Motor symptoms were similar in individuals with normal and abnormal scans. Olfactory testing was concordant with imaging in 90% cases.
Tachibana et al. (2017) [65]	9 patients with DIP	Neurological signs and imaging may be useful to diagnose early stages of unmasked PD in individuals with a previous diagnosis of DIP.
Gajos et al. (2019) [66]	11 patients with DIP and asymmetric symptoms	The authors calculated the indices for the whole striatum, putamen, caudate, and putamen/caudate ratio. They did not find significant differences in radiotracer uptake in structures contralateral to more severe clinical symptoms when compared to the homolateral hemisphere.
Aamodt et al. (2022) [67]	34 US Veterans with DIP	Imaging was abnormal in 35%. Compared to normal, the individuals with abnormal imaging had gait impairment, hyposmia, and non-motor symptoms.

Abbreviations: DIP, drug-induced parkinsonism; PD, Parkinson’s disease; SPECT, single-photon emission computerized tomography; UPDRS, Unified Parkinson’s Disease Rating Scale; and VP, vascular parkinsonism.

**Table 8:** Labeled indications for [18F]-DOPA.

Agency	Descriptor
European Medicines Agency	This medicinal product is for diagnostic use only. Fluorodopa (18F) is indicated for use with positron emission tomography (PET) in adults and paediatric population.
	Neurology PET with fluorodopa (18F) is indicated for detecting the loss of functional dopaminergic neuron terminals in the striatum. It can be used for the diagnosis of Parkinson’s disease and differentiation between essential tremor and parkinsonian syndromes. To help differentiate probable dementia with Lewy bodies from Alzheimer’s disease. DaTSCAN is unable to discriminate between dementia with Lewy bodies and Parkinson’s disease dementia.
US Food and Drug Administration	Fluorodopa F 18 Injection is a radioactive diagnostic agent indicated for use in positron emission tomography (PET) to visualize dopaminergic nerve terminals in the striatum for the evaluation of adult patients with suspected Parkinsonian syndromes (PS). Fluorodopa F 18 PET is an adjunct to other diagnostic evaluations.

**Table 9:** Comparison among different neuroimaging techniques for the differentiation between PD and DIP.

Neuroimaging	Pros	Cons
Dopamine radiotracers	Less confounding factors than others scintigraphy studies	Invasive, scintigraphy study
[18F]-FDG	Disease-related pattern	Invasive, scintigraphy study. A significant number of false-positive results
[123I]-MIBG	Cardiac abnormalities are found before striatal region lesions in PD	Invasive, scintigraphy study
MRI (swallowtail appearance)	Non-invasive	15% of the patients with DIP will have abnormal results
Transcranial ultrasound	Non-invasive, high specificity	Sensitivity depends on the exact cut-off value of the area being analyzed
Optical coherence tomography	Non-invasive	There is no benefit in early stage of DIP

## Data Availability

Data are contained within the article.
